# Investigation of a novel approach to scoring Giemsa-stained malaria-infected thin blood films

**DOI:** 10.1186/1475-2875-7-62

**Published:** 2008-04-21

**Authors:** Owen Proudfoot, Nathan Drew, Anja Scholzen, Sue Xiang, Magdalena Plebanski

**Affiliations:** 1 Burnet Institute, Austin Hospital campus, Studley Road, Heidelberg, Melbourne, 3084, Australia; 2 Independent computing advisor, Melbourne, Australia; 3 Department of Immunology, Monash University, Melbourne, Australia

## Abstract

Daily assessment of the percentage of erythrocytes that are infected ('percent-parasitaemia') across a time-course is a necessary step in many experimental studies of malaria, but represents a time-consuming and unpopular task among researchers. The most common method is extensive microscopic examination of Giemsa-stained thin blood-films. This study explored a method for the assessment of percent-parasitaemia that does not require extended periods of microscopy and results in a descriptive and permanent record of parasitaemia data that is highly amenable to subsequent 'data-mining'. Digital photography was utilized in conjunction with a basic purpose-written computer programme to test the viability of the concept. Partial automation of the determination of percent parasitaemia was then explored, resulting in the successful customization of commercially available broad-spectrum image analysis software towards this aim. Lastly, automated discrimination between infected and uninfected RBCs based on analysis of digital parameters of individual cell images was explored in an effort to completely automate the calculation of an accurate percent-parasitaemia.

## Background

When conducting blood-stage malaria trials involving live malaria challenges in mice, frequent assessment of their 'percent parasitaemia' is necessary throughout the duration of the experiment. Similarly, the *in vitro *expansion of the human malaria parasite *Plasmodium falciparum *requires frequent monitoring of the percent parasitaemia of cultures [1]. While FACS-based experimental methods have been reported [2,3], the most common method used to assess percent parasitaemia remains extensive microscopic scrutiny of Giemsa-stained thin blood-films. Lengthy sessions of microscopy are tedious and uncomfortable though, thus this is not a popular task among researchers. Particularly where new models are being explored by a laboratory or future data scrutiny or mining may be required, retention of all thin-blood-films is desirable. The slides can become a storage burden and degrade over time however. Additionally, they do not represent a record of the exact microscopic-field from which the data was derived at the time of recording.

Initially, this study sought to improve the qualitative substance of 'percent parasitaemia' data utilizing a combination of microscopy, digital photography and computer software. A simple programme was written towards this aim in the 'Visual-Basic [4]' computer language. Once a digital image of the Giemsa-stained blood-film is captured, the programme enables the researcher to 'score' each cell from a comfortable distance on a monitor via mouse clicks, rather than while looking down a microscope. Every decision at the cell level is recorded via a small colour-coded addition to the on-screen image. This approach negates long microscopy sessions and the possibility of cells inadvertently being included twice ('double-counts') in the assessment of percent-parasitaemia.

Optional parameters include scoring a lymphocyte count per microscopic-field, subdivision of uninfected RBCs based on maturity, and subdivision of malaria-infected RBCs based on parasite-stage. Regardless of the parameters chosen, overall percent-parasitaemia is calculated as scoring data are entered. Via this approach, the microscopy and scoring need not be performed by the same researcher, and the data-base created represents a legitimate substitute for actual blood-film-slides, which is more amenable to long term storage and future scrutiny. The programme ('Plasmoscore') is freely available for download [5] as a stand alone programme.

After development of a simple programme affording a permanent digital record of the precise derivation of parasitaemia data, partial automation of the process via customizing commercially available digital-image analysis software (Image-Pro^® ^[6]) was attempted. A 'macro' combining an automated total cell count for each microscopic field with manual user determination of each infected cell was developed. Lastly, automated discrimination between infected and uninfected cells was attempted in an effort to completely automate the determination of the percent-parasitaemia depicted in digital thin-blood-film images.

## Results

### Manual scoring of a digital image (*Plasmoscore*)

**Figure 1:**
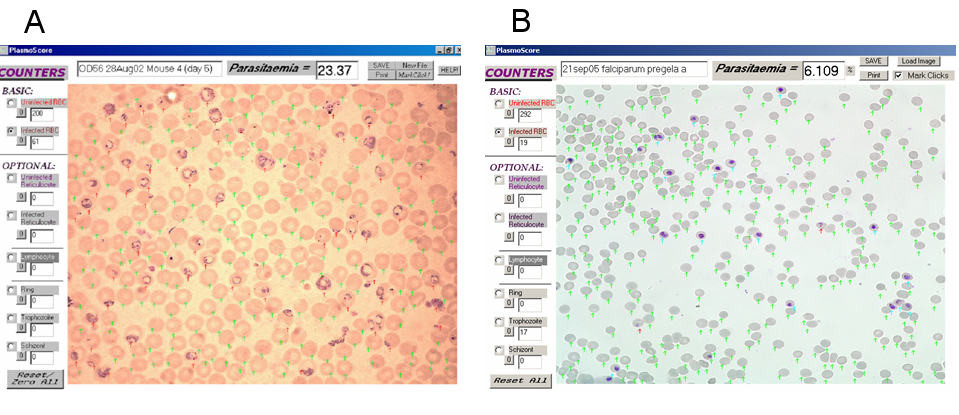
**Screen-shots of Giemsa-stained thin-films of malaria-infected murine and human blood scored using the freely available '*Plasmoscore' *software**. **a**. A scored image from a C57BL/6 mouse infected with *Plasmodium yoelii *YM, captured at 1000× magnification. Green arrows indicate unifected RBCs, and red arrows indicate infected RBCs. In this example the user has opted not to discriminate between the different stages of infected RBC. **b**. A scored image of a human RBC culture experimentally infected with *P. falciparum*, captured at 600× magnification. In this example the user has chosen to specifically determine the number of trophozoites present (blue arrows), and infected cells at any other stage of development have simply been scored as 'infected RBC' (red arrows).

The *Plasmoscore *programme was written in the Visual-Basic [4] computer language. Upon execution of the programme, the user is prompted to load a digital image of a Giemsa-stained thin blood film. The user then selects which of a series of cell types (infected RBC, uninfected RBC, ring infected RBC etc) they will score, and clicks every cell in the image that falls into this category. Each decision made by the user is recorded via a small colour-coded mark superimposed onto the image (Figures 1a and 1b). After scoring all desired cell-types, a total 'percent parasitaemia' is calculated, and this and the scored image can be saved as a permanent record.

### Semi-automated scoring of a digital image (*Image-pro*^®^)

**Figure 2:**
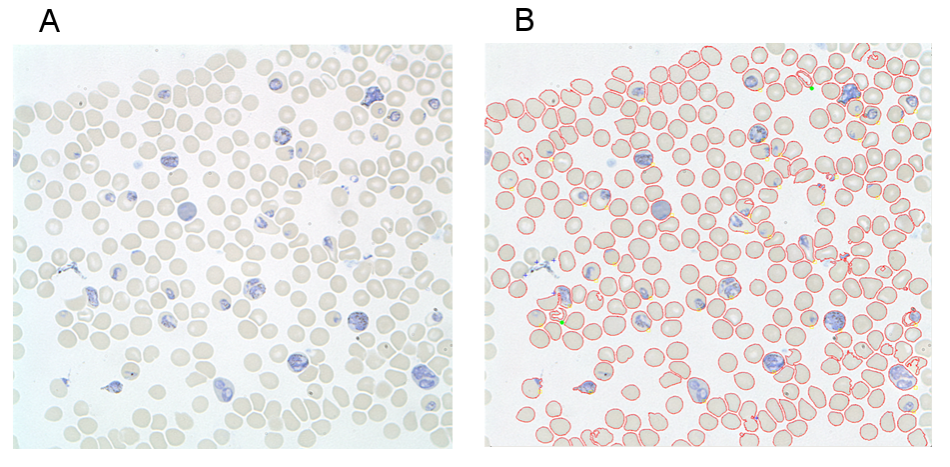
**Screen shots of data derived from a *P. yoelii *infected thin blood film before and after partially automated determination of percent-parasitaemia**. **a**. A section of a blood film derived from a *P. yoelii *YM infected C57BL/6 mouse. **b**. The same section after automatic total cell count, user correction thereof and parasitaemia scoring; blue crosses indicate where the user has added cells to the total cell-count, and green dots indicate where the user has subtracted cells. Yellow circles indicate cells that the user has deemed infected.

**Figure 3:**
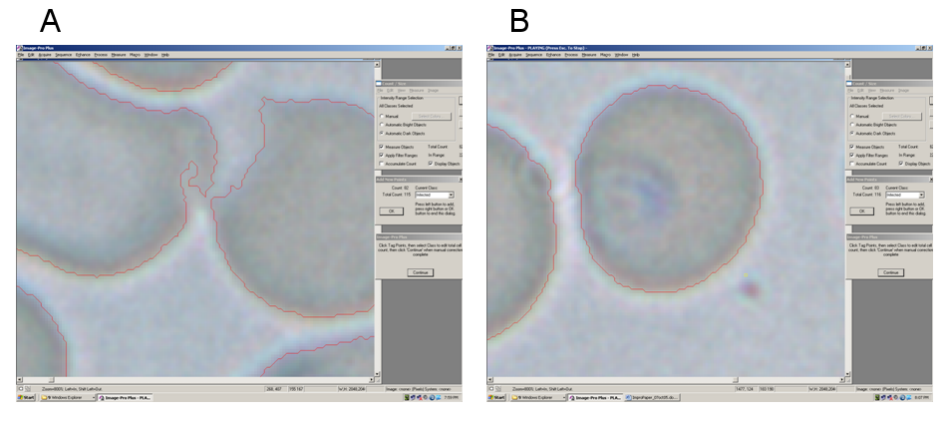
**Screen shots depicting utilisation of the mouse-wheel-linked 'enhanced magnification' (zoom) function to aid accurate scoring**. **a**. The user has 'zoomed in' on two potentially agglutinated cells to an ultimate magnification of 16000×, to confirm that the software has accurately identified them as two separate cells. **b**. The user has zoomed in on a cell that is ambiguous re infection status under standard magnification, and confirmed that it is infected.

A commercially available image-analysis software package, 'Image-Pro' [6], was explored as a vehicle for accurate automation of a total cell count. A macro imposing a standard series of manipulations to the digital images loaded including hue, sharpness, contrast and background-flattening adjustments was generated. The series of adjustments yields an image of which the software can accurately determine the total number of cells depicted therein, automatically (Figure 2a). Boundaries are then superimposed around each individual cell (Figure 2b). At this point the user can scrutinize the automated total cell count (utilizing a zoom function if necessary) and correct any inaccuracies via a mouse click (Figure 3a). Once an accurate total cell count has been determined, the user clicks on individual infected cells in the blood-film. The zoom-function can also be utilized at this point to afford closer examination of cells that are ambiguous re infection-status (Figure 3b). The percent parasitaemia of the film is thus calculated semi-automatically, via an analysis sequence safe-guarded by user scrutiny at each crucial stage. All data, including the total cell count before and after correction, infected cell count, percent parasitaemia and associated image files are automatically exported and saved to a running excel file.

**Figure 4:**
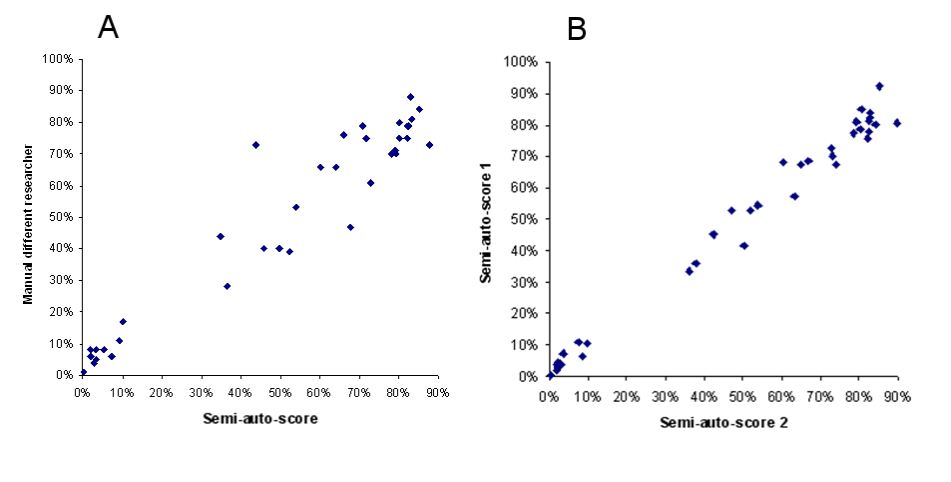
**Comparison of manually and semi-automatically scored malaria infected blood-films**. **a**. Scores yielded by semi-automated percent-parasitaemia plotted against scores derived from the same slide via manual scoring by a different researcher (correlation = .96). **b**. Semi-automated scores of two images derived from different areas of the same slide, analysed by the same researcher, plotted against each other (correlation = .99).

The reliability and reproducibility of percent-paracitaemia-data obtained via this method was formally assessed using archival blood-films from mice that had been experimentally infected with malaria and assessed via the traditional method. In an effort to gage the validity of semi-automated parasitaemia determination as compared to entirely manual percent-parasitaemia assessment, semi-automated parasitaemia scores were compared with those derived from entirely-manual assessment of the same blood-film by a different researcher (Figure 4a). Internal (sample) reproducibility using the method was gauged by comparing semi-automated percent-parasitaemias derived from images of different areas of the same slide, assessed by the same researcher (Figure 4b).

### Completely automated scoring of a digital image

**Figure 5:**
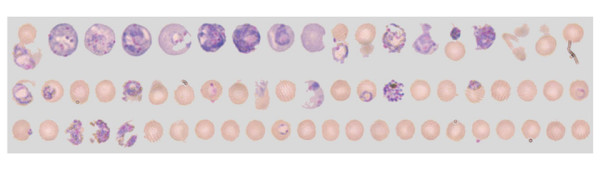
The 'interphase' after automatic identification of individual cells and gridding of these ranked on various features potentially discriminating infected and uninfected cells.

Attempts were made to identify a combination of the parameters of isolated objects (in this case 'infected cells') including size, internal-contrast and brightness, in order to automatically discriminate infected from uninfected RBCs. Based on such parameters, cells were 'ranked' and organized into a grid via a macro, based on the likelihood that they were infected. While 'obviously infected' and 'obviously uninfected' cells were accurately discriminated via this approach, the interphase of the ranked-grid contained sustantial overlap (Figure 5), necessitating substantial user correction.

## Discussion and conclusion

There are several advantages conferred by using digital image based methods of scoring thin-blood-films for percent-parasitaemia over the traditional method. Foremost, large numbers of films can be scored without extended hours of microscopy. Automating the total cell count via the above-described method substantially reduces the time taken to score each film; if parasitaemia appears low (<50%) then the user need only score infected cells, and if it appears high the user need only score uninfected cells, to derive the over-all percent-parasitaemia. The graphic nature of the permanent records generated enable data of interest to be perused by multiple researchers simultaneously, emailed to distant researchers or retrospectively 'mined' for additional details such as differential parasite-stage analysis and lymphocyte counts. Unlike an actual blood-film, the digital image does not degrade over time and is not an incumbrance to store or transport.

Monetary considerations limit the utilisation of this approach. The most obvious is the requirement of a high-resolution, wide lens, microscope-mounted digital camera. At the operational level if the camera used cannot capture at least 400 cells per image, multiple images per blood-film will be required and the programme will need to combine user input appropriately to derive a valid percent parasitaemia per sample. Commercial software was used to enable an automatic cell-count, which would represent a financial limitation for many laboratories. Notably, there are various free 'open-source' software projects in a constant state of development such as NIH-image [7] and Image-J [8], which may prove useful to this end in the future.

While it was found that accurate automated determination of the total-cell-count of Giemsa-stained thin-blood-film images was ultimately achievable via commercial software [6], the success of this was affected by the uniformity with which thin blood-films were generated, processed and photographed. Particularly, thickly spread, over-fixed or over-stained blood films were not accurately processed by automated cell-counting software.

The ultimate aim of completely automated scoring of malaria infected thin-blood film images was explored by the authors, but remains elusive. Attempts were made to utilize parameters such as background flattening, internal-contrast and brightness of isolated objects (in this case 'cells') to discriminate infecteds from uninfecteds. Various factors including the presence of lymphocytes and the increased incidence of reticulocytes hampered these efforts however, resulting in a requirement for extensive 'user-correction'. The automated generation of a graphic grid 'ranking and grouping' cells based on a combination of criteria (predicting the likelihood that cells were infected) was achieved, which could substantially reduce the user-time required to score cells as infected or uninfected. It is anticipated that digital imaging processes will be widely utilized to determine the percent parasitaemia of malaria infected thin blood-films in the future. The extent to which this can be automated will rely on the adaptability of the software utilized. Given the complexity of the task, completely accurate yet entirely automated (no user correction required) determination of the process seems distant.

## Competing interests

The author(s) declares that they have no competing interests.

## Authors' contributions

OP: Created the project and conceptualised the idea of automated scoring via software, involved in the generation, integration and testing of computational and laboratory methods at all levels.

ND: Principal computing advisor, conceptualised the idea of automated scoring via software, generated original computer programmes (written in visual basic) and wrote all 3^rd ^party macros for the image analysis software package Image-Pro^®^, involved in ongoing testing throughout the project.

AS: Generated and tested malaria infected human blood samples utilizing the methods described herein, providing evidence the novel scoring method was useful in the context of human malaria research.

SX: Principal microscopy advisor, introduced and formed the conceptual idea of using the image analysis software package Image-Pro^® ^to the project for generating an automated scoring system.

MP: Facilitated the ongoing development of the project from it's inception via the provision of practical resources.

All authors have read and approved the final manuscript
